# Microbiome, metabolome, and transcriptome analyses in esophageal squamous cell carcinoma: insights into immune modulation by *F*. *nucleatum*

**DOI:** 10.1093/procel/pwae063

**Published:** 2024-10-29

**Authors:** Xue Zhang, Jing Han, Yudong Wang, Li Feng, Zhisong Fan, Yu Su, Wenya Song, Lan Wang, Long Wang, Hui Jin, Jiayin Liu, Dan Li, Guiying Li, Yan Liu, Jing Zuo, Zhiyu Ni

**Affiliations:** Department of Oncology, The Fourth Hospital of Hebei Medical University, Shijiazhuang 050011, China; Department of Oncology, The Fourth Hospital of Hebei Medical University, Shijiazhuang 050011, China; Department of Oncology, The Fourth Hospital of Hebei Medical University, Shijiazhuang 050011, China; Department of Oncology, The Fourth Hospital of Hebei Medical University, Shijiazhuang 050011, China; Department of Oncology, The Fourth Hospital of Hebei Medical University, Shijiazhuang 050011, China; Department of Oncology, The Fourth Hospital of Hebei Medical University, Shijiazhuang 050011, China; Department of Oncology, The Fourth Hospital of Hebei Medical University, Shijiazhuang 050011, China; Department of Oncology, The Fourth Hospital of Hebei Medical University, Shijiazhuang 050011, China; Department of Oncology, The Fourth Hospital of Hebei Medical University, Shijiazhuang 050011, China; Department of Oncology, The Fourth Hospital of Hebei Medical University, Shijiazhuang 050011, China; Department of Oncology, The Fourth Hospital of Hebei Medical University, Shijiazhuang 050011, China; Department of Oncology, The Fourth Hospital of Hebei Medical University, Shijiazhuang 050011, China; Department of Nephrology, The Key Laboratory of Basic Research on Blood Purification Application in Hebei Province, Affiliated Hospital of Hebei Engineering University, Handan 056002, China; Department of Oncology, The Fourth Hospital of Hebei Medical University, Shijiazhuang 050011, China; Department of Oncology, The Fourth Hospital of Hebei Medical University, Shijiazhuang 050011, China; Oncology Center, Affiliated Hospital of Hebei Engineering University, Handan 056002, China; Clinical Medical College, Hebei University of Engineering, Handan 056038, China; Central Laboratory, Hebei Collaborative Innovation Center of Tumor Microecological Metabolism Regulation, Affiliated Hospital of Hebei University, Baoding 071000, China

**Dear Editor,**

Esophageal cancer is the seventh most common malignant tumor in China and has the third highest fatality rate worldwide (Siegel et al., 2022). The most common type in China is esophageal squamous cell carcinoma (ESCC), which has shown a promising response to immune checkpoint inhibitors (Doki et al., 2022; Sun et al., 2021). However, challenges remain in effectively using immunotherapy due to varying patient responses and difficulties in identifying suitable candidates.

The esophagus serves as the vital link between the oral cavity and the stomach. Its unique anatomical position renders it susceptible to various microorganisms. This exposure significantly heightens the risk of intra-tumoral microbiota colonization in ESCC. Long-term dental caries and chronic periodontitis have been linked to immunotherapy resistance in ESCC. This resistance could be attributed to persistent streptococcal infections in the oral and pharyngeal regions (Gao et al., 2018). Furthermore, metabolites originating from gut microbiota play a critical role in mediating communication between microbiota and the host immune system (He et al., 2021; Mager et al., 2020; Wang et al., 2022). Microorganisms possess the ability to modulate the sensitivity of ESCC to immunotherapy (Mager et al., 2020; Matson et al., 2018). Recent research unequivocally demonstrated that the intra-tumoral microbiota plays a crucial role in influencing tumor responses to immunotherapy (Lo et al., 2021; Wu et al., 2023). However, the mechanistics are still not fully elucidated.

We conducted a comprehensive analysis of microbiome, metabolome, and transcriptome analyses of 52 ESCC patients, including 43 untreated and 9 treated patients (Fig. 1A; Supplementary Materials and Methods). Figure S1A displayed preoperative gastroscopy images and corresponding postoperative hematoxylin-eosin staining images, and 16S rRNA microbiota analysis of cohort 1 at the genus level revealed distinct differences between untreated ESCC and paired normal tissues, including *Fusobacterium* (Fig. S1B and S1C). LEfSe analysis identified *p_Fusobacteria*, *c_Fusobacteriia*, and *o_Fusobacteriales* as dominant germs in ESCC tissues, with untreated tissues showing higher levels of *f_Fusobacteriaceae* than treated ones (Figs. 1B and S1C). Higher levels of *Fusobacterium* were observed in the advanced stage (Fig. S1D and S1E) but decreased significantly in the treated cases (Fig. 1D), particularly in patients undergoing chemo-immunotherapy compared with chemotherapy alone (Fig. 1E). In our previous studies, oral mucosal and saliva samples were collected from ESCC patients undergoing immunotherapy (Zuo et al., 2023). Further analysis revealed that patients who achieved clinical benefit had lower levels of *Fusobacterium* in their oral samples compared with those who with no clinical benefit (Fig. S1F). Analyzing *Fusobacterium* alone reaffirmed its significant overexpression in ESCC tissue (Fig. 1F). Elevated *Fusobacterium* abundance correlated with worse TNM and N stages, exhibiting significant downregulation post-treatment (Fig. 1G–I).

**Figure 1.**
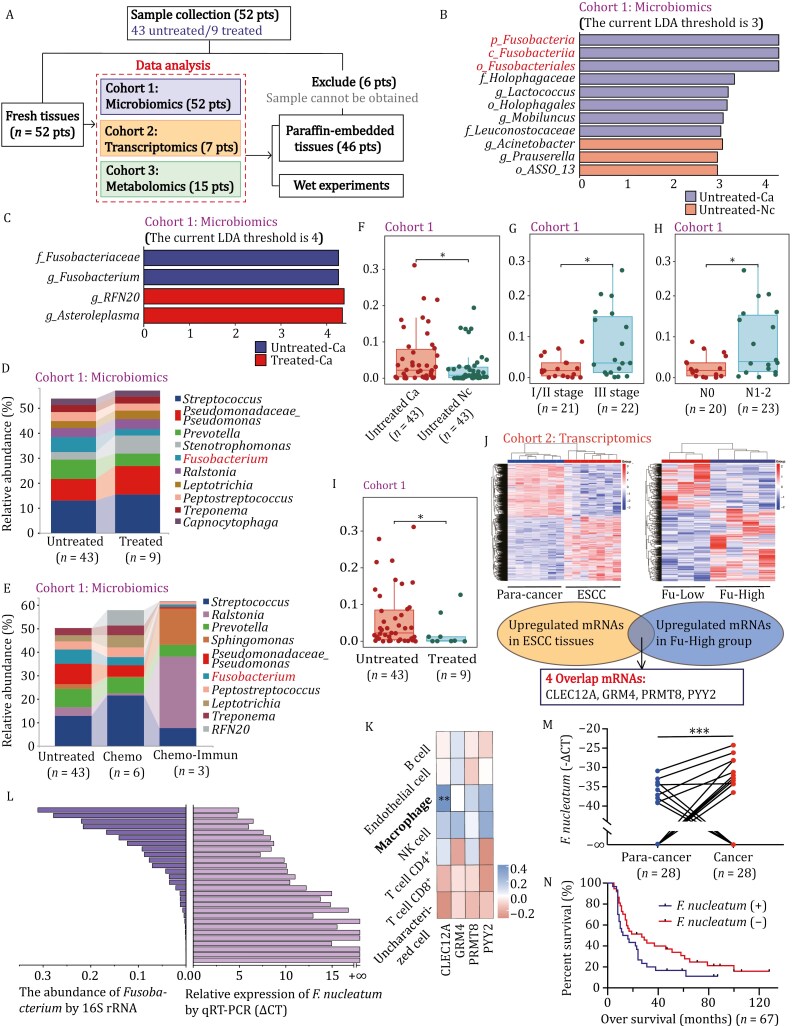
***Fusobacterium nucleatum*** is highly expressed in ESCC tissue and may be associated with immune response. (A) Overview of the study design. (B and C) LEfSe analysis of differential species (Wilcoxon test). (D and E) Species composition analysis of samples under different grouping conditions. Data were processed based on the mean values within each group. (F) Abundance of *Fusobacterium* genus in untreated ESCC and normal tissues (*P *= 0.046). (G and H) Abundance of *Fusobacterium* genus in untreated ESCC with different TNM (*P *= 0.045) and *N* stage (*P *= 0.038). (I) Abundance of *Fusobacterium* genus in untreated and treated ESCC tissues (*P *= 0.046). (J) Screening of oncogenes associated with *Fusobacterium* abundance in ESCC tissues. (K) The correlation heatmap between the four mRNAs and immune score based on TCGA data related to Asian ESCC analysis. Different colors represent different relationships (positive or negative), the darker the color, and the stronger the relation. Asterisks stand for significance levels. (L) The abundance of *F*. *nucleatum* and *Fusobacterium* in the same 28 ESCC tissue specimens from cohort 1. Data of *Fusobacterium* were obtained from 16S rRNA sequencing, while the abundance of *F*. *nucleatum* was tested by qRT-PCR. (M) The abundance of *F*. *nucleatum* of gene chip containing ESCC and para-cancer tissues. (N) Overall survival analysis of ESCC with or without *F. nucleatum* infection. ^*^P < 0.05, ^**^P < 0.01, ^***^P < 0.001.

ESCC tissues in cohort 2 were categorized into Fu-Low group and Fu-High group based on *Fusobacterium* abundance. There were four mRNAs not only upregulated in ESCC compared with para-cancer tissues but also in Fu-High compared with Fu-Low: *C-Type Lectin Domain Family 12 Member A* (*CLEC12A*), *Glutamate Metabotropic Receptor 4* (*GRM4*), *Protein Arginine Methyltransferase 8* (*PRMT8*), and *Peptide YY 2* (*PYY2*) (Fig. 1J). A correlation analysis between *Fusobacterium* abundance and the target gene expression was conducted in ESCC samples (Fig. S2A). Gene Ontology analysis on differentially expressed genes (DEGs) based on *Fusobacterium* abundance revealed associations with inflammatory response pathways (Fig. S2B). Human multi-database association annotation analysis on Fu-High and Fu-Low DEGs provided insights into immune-related characteristics (Fig. S2C). Subsequently, we conducted an immune correlation analysis with data sourced from TIMER database. The corresponding clinical information of Asian ESCC patients was showed in Table S1. Results indicated a notable positive association between *CLEC12A* and macrophages (Fig. 1K), as well as the respective genetic markers (Fig. S2D and S2E). Immunofluorescence (IF) staining indicated that compared with para-cancer tissues, the presence of tumor-associated macrophages (TAMs) in ESCC tissues increased. Moreover, we observed an interesting phenomenon that the number of TAMs in treated ESCC tissue increased compared with untreated ESCC tissue (Fig. S2F). Besides, there were significant downregulation of M1-type macrophage-associated genes (*IL-12A*, *CD68*, *CXCL12*, *IRF5*) and upregulation of M2-type macrophage-associated genes (*TGFB1*, *TGFB2*, *IL10*, *CCL18*) in ESCC (Fig. S2G). The joint analysis revealed that chemokines associated with *Fusobacterium* abundance include *CCR8*, *CXCL1*, and *CXCL8* (Fig. S2H). We hypothesized that *Fusobacterium*’s target genes are intricately associated with immune responses and may exhibit a specific relationship with TAMs.

*Fusobacterium nucleatum* was the represented species in *Fusobacterium* genus. To explore whether *F*. *nucleatum* plays a pivotal role as well as *Fusobacterium*, a quantitative real-time polymerase chain reaction (qRT-PCR) analysis was performed on 28 ESCC tissues of cohort 1. The results revealed that the expression pattern of *F*. *nucleatum* closely mirrored the abundance trend within the *Fusobacterium* genus (Fig. 1L). Besides, it demonstrated significantly higher expression levels of *F*. *nucleatum* in ESCC tissues compared with para-cancer tissues (Fig. 1M). We performed qRT-PCR on a gene chip containing 67 ESCC tissues and their corresponding 28 adjacent normal tissues. Figure S3A displayed the clinical and pathological characteristics. It was found that *F. nucleatum* infection is linked to worse TNM staging (Fig. S3B), and a poorer prognosis than non-*F*. *nucleatum* infection (log‐rank *P* = 0.086, Fig. 1N), which was consistent with previous research (Ding et al., 2023; Yamamura et al., 2016). However, the difference was not statistically significant, likely due to limited cases.

In order to elucidate the association between *F*. *nucleatum* and immune cells, fluorescent probes of *F*. *nucleatum* and IF staining of immune cells were performed on paraffin-embedded specimens. It was found that in areas with *F*. *nucleatum* infection, the number of TAMs was higher (Fig. 2A) and the number of CD8^+^ T cells is relatively low, while no significant change in CD4^+^ T cells (Fig. S3C). Database analysis also showed a significant positive correlation between *CLEC12A* and TAMs but no significant correlation with other immune cells (Fig. S3D). Dual IF results from ESCC paraffin tissue revealed differences in TAMs polarization based on *F*. *nucleatum* infection states (Figs. 2B and S3E). Despite the limited number of specimens, these results supported our hypothesis that *F*. *nucleatum* may exhibit a specific relationship with TAMs.

**Figure 2.**
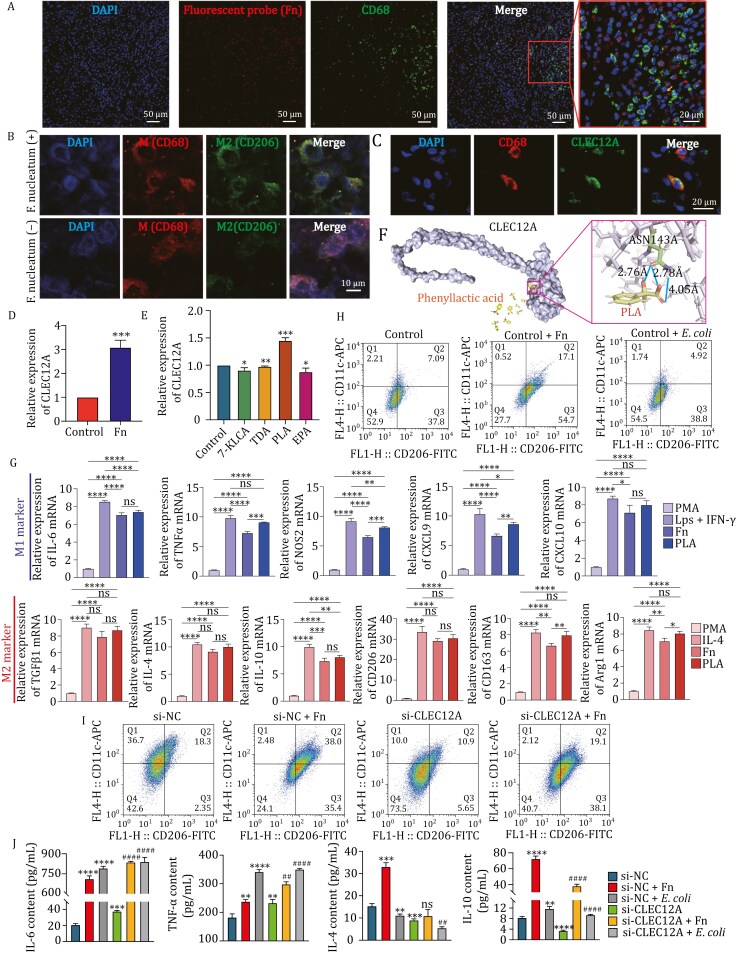
The effects of ***F. nucleatum*, PLA, and CLEC12A on macrophages**. (A) Fluorescent probes of *F*. *nucleatum* and IF staining of CD68 on ESCC paraffin embedded specimens. (B) Dual IF of CD68 and CD206 in ESCC tissues with or without *F*. *nucleatum* infection. (C) Dual IF of CD68 and CLEC12A. (D) The change of mRNA expression of CLEC12A in THP1 macrophages after co-culture with *F*. *nucleatum*. (E) The change of mRNA expression of CLEC12A in THP1 macrophages after treated with metabolites. (F) The structural prediction models of PLA and CLEC12A protein exhibited stability in Rosetta scoring, with a van der Waals binding energy of −4.468, an electrostatic interaction score of −4.468, and a total energy score of −974.412. Visualization of the model was conducted using PyMOL software, confirming that the 143rd asparagine residue of the A chain of the CLEC12A protein interacts with phenylalanine. (G) mRNA expression of different markers of M1 and M2 macrophage in different groups. ^ns^*P >* 0.05, ^*^P < 0.05, ^**^P < 0.01, ^***^P < 0.001, ^****^P < 0.0001. (H) Flow cytometry detection of changes in the distribution of macrophage CD11c and CD206 after co-culture with *F*. *nucleatum* or *E*. *coli*. (I) Flow cytometry detection of changes in the distribution of M1 and M2 macrophage in different groups. (J) ELISA test of the cytokines in different group of cell culture supernatant. ^**^P < 0.01, ^***^P < 0.001, ^****^P < 0.0001 vs. si-NC; ^##^*P *< 0.01, ^####^*P *< 0.0001, ^ns^*P >* 0.05 vs. si-CLEC12A.

CLEC12A belongs to the myeloid C-type lectin receptor, which is a pattern recognition receptor for bacteria and is widely expressed on immune cells, including macrophages. Multiple IF staining reveals co-localization of CLEC12A and CD68 (Fig. 2C). Notably, *F*. *nucleatum* was present both inside and outside TAMs (Fig. 2A). Subsequent electron microscopy analysis of samples from co-cultured THP1 macrophages with *F. nucleatum* at a ratio of 1:100 revealed a limited presence of the bacterium within cells (Fig. S4A). This suggested that *F*. *nucleatum* may impact TAMs either directly or indirectly, with metabolites potentially playing a significant role in this interaction. Then, we conducted non-targeted metabolomics on cohort 3. There was a significant difference in metabolites between ESCC and normal tissues, encompassing untreated and treated patients (Fig. S4B). For untreated patients, 66 upregulated and 27 downregulated metabolites were observed in ESCC tissue, while for treated patients, 3 upregulated and 22 downregulated metabolites were identified, indicating treatment-associated changes (Fig. S4C). Kyoto Encyclopedia of Genes and Genomes enrichment analysis highlighted the involvement of numerous metabolites in linoleic acid metabolism which is a kind of free fatty acids (FFA) (Fig. S4D and S4E). Cluster analysis disclosed 12 *Fusobacterium*-associated metabolites, also including FFA (Fig. S4F). OPLS-DA analysis demonstrated eight upregulated and four downregulated metabolites in the Fu-High group (Fig. S4G). Integrating metabolite and microbiological data also identified FFA correlating with *Fusobacterium* (Fig. S4H), suggesting that *Fusobacterium* is crucial in FFA metabolism. Subsequently, a targeted FFA metabolomics analysis was performed on the cell supernatant of KYSE-30 and ECA109, which confirmed elevated levels of metabolites in *F*. *nucleatum* co-cultured groups (Table S2). Finally, 4 candidate metabolites were selected: Tridecanoic acid (TDA), Eicosapentaenoic acid (EPA), 7-Ketolithocholic acid (7-KLCA), and Phenyllactic acid (PLA).

Then, we co-cultured *F*. *nucleatum* with THP1 macrophages for wet experiments. There was a significant increase of *CLEC12A* after co-culturing with *F*. *nucleatum* (Fig. 2D). The activity of THP1 macrophages changed following the interventions of TDA, EPA, 7-KLCA, and PLA (Fig. S5A). Then, the intervention concentration was set at 10 μmol/L for EPA, and 500 nmol/L for 7-KLCA, PLA, and TDA. Specifically, the intervention of THP1 macrophages with PLA showed upregulation of *CLEC12A*, which was consistent with *F*. *nucleatum* (Fig. 2E). To investigate the interaction between PLA and the membrane protein CLEC12A, we obtained structural models of PLA and the CLEC12A protein from the PubChem and UniProt databases, respectively. Docking experiments were conducted using the Rosetta software. The target model exhibited stability in Rosetta scoring. Subsequently, the model was uploaded to the PLIP network server. The results revealed a well-defined region of strong hydrogen bonding interaction between protein side chain atoms (Num: 2350) and ligand atoms (Num: 4324). The experiment ultimately confirmed that PLA interacts with the 143rd asparagine residue of the A chain of the CLEC12A protein (Fig. 2F). Subsequently, we conducted an analysis of specific markers associated with M1 and M2 macrophages, utilizing LPS combined with IFN-γ as the positive control for M1 polarization and IL-4 as the positive control for M2 polarization in THP-1 macrophages. The results indicated that treatment with *F*. *nucleatum* or PLA significantly enhanced M1 polarization, as evidenced by a marked increase in the expression levels of the *IL-6*, *TNF-α*, *NOS2*, *CXCL9*, and *CXCL10* genes. Meanwhile, both *F*. *nucleatum* and PLA interventions in THP-1 macrophages enhanced M2 polarization, as evidenced by a marked increase in the expression levels of the *TGF-*β*1*, *IL-4*, *IL-10*, *CD206*, *CD163*, and *Arg1* genes, with PLA having a stronger effect. Notably, the M2 marker *CD206* gene showed M2 anti-inflammatory activity (nearly 30-fold increase) significantly more than M1 pro-inflammatory activity (about 8-fold increase) (Fig. 2G). Flow cytometry results indicated a significant increase in M2-type THP-1 macrophages (both CD206 and CD163) after co-culturing with *F*. *nucleatum*, whereas no such change was detected when co-cultured with *Escherichia coli* (Figs. 2H and S5B). Furthermore, PLA, in contrast to the other three metabolites, also induced an upward trend in M2 macrophage levels (Fig. S5C).

Subsequently, we devised a rescue experiment to investigate the effects of *F*. *nucleatum* and *CLEC12A* on the polarization of THP1 macrophages. Our findings indicated that *CLEC12A* knockdown not only resulted in a reduction in the proportion of M2 macrophages but also partially mitigated the increase in M2 macrophage levels induced by *F*. *nucleatum* infection (Figs. 2I and S5D). The ELISA assay revealed that *F*. *nucleatum* infection lead a significant increase in IL4 and IL10, and knocking down *CLEC12A* significantly reduced IL4 and IL10 compared with the control group. Interestingly, THP1 macrophages with low *CLEC12A* expression did not demonstrate an elevation in IL4 following *F*. *nucleatum* infection. Furthermore, *CLEC12A* knockdown resulted in an enhanced secretion of pro-inflammatory cytokines IL-6 and TNF-α in THP1 macrophages (Fig. 2J). These findings indicated that CLEC12A potentially plays a pivotal role in the *F*. *nucleatum*-mediated inflammatory response of macrophages.

In conclusion, this study presented a novel integration of microbiome, metabolome, and transcriptome to investigate the metabolites and target genes associated with *F*. *nucleatum*. The findings suggested a correlation between *F*. *nucleatum* infection and immune characteristics in ESCC, with particular emphasis on TAMs. *Fusobacterium nucleatum* not only upregulated M1 markers in THP-1 macrophages but also significantly elevates M2-specific markers, with the most notable increase seen in the anti-inflammatory marker *CD206*. *Fusobacterium nucleatum* and its associated metabolite, PLA, upregulated the expression levels of *CLEC12A* in THP-1 macrophages, which is essential for the induction of the CD206 and CD163 phenotypes by *F*. *nucleatum*. We hypothesize that *F*. *nucleatum* may activate and polarize macrophages, affecting the tumor immune microenvironment. Further research is needed to understand these mechanisms and develop new immunotherapy strategies.

## Supplementary data

Supplementary data is available at *Protein & Cell* online at https://doi.org/10.1093/procel/pwae063.

pwae063_suppl_Supplementary_Figures_S1-S5_Table_S2-S3

pwae063_suppl_Supplementary_Tables_S1

## Footnotes

We thank Shanghai Personal Biotechnology Co., Ltd. (Shanghai, China) for technical support. This work was supported by the Natural Science Foundation of China (Grant No. 82373072) awarded to Z.N., the Key Project of Hebei Province Natural Science Precision Medicine Joint Fund (H2022201067) awarded to Z.N., and the Natural Science Foundation of Hebei Province (Grant No. H2024206159) awarded to J.Z.

The authors declare that there is no conflict of interest. This study was approved by the Ethical Review Boards of the Fourth Hospital of Hebei Medical University (Ethics Approval Number: 2023KS248). Informed consent was obtained from all individual participants included in the study.

All authors have read and approved the final manuscript and consent to its publication. The raw data of 16S rRNA and transcriptomics performed in this study have been uploaded to the Sequence Read Archive database (PRJNA1025372 and PRJNA1053886).

X.Z. and J.Z. conceived the study and supervised the project. L.F., Z.S.F., L.W., and Y.D.W. collected the samples. L.W., H.J., and J.Y.L. analyzed the data. X.Z., J.Z., and J.H. designed the experiments. Y.S., D.L., Y.W.S., G.Y.L., and Y.L. carried out the experiments. X.Z., J.H., and Y.D.W. wrote and proofread the manuscript. J.Z. and Z.Y.N. provided the research funding. All authors read and approved the final manuscript.
