# Supplementary material for: Microbiome, metabolome, and transcriptome analyses in esophageal squamous cell carcinoma: insights into immune modulation by F. nucleatum
Source: Protein Cell. 2024 Oct 29;16(6):491–6. doi: 10.1093/procel/pwae063 (PMC12187192; doi:10.1093/procel/pwae063)
Supplement: pwae063_suppl_Supplementary_Figures_S1-S5_Table_S2-S3 [file pwae063_suppl_supplementary_figures_s1-s5_table_s2-s3.pdf]

Supplementary figure 1

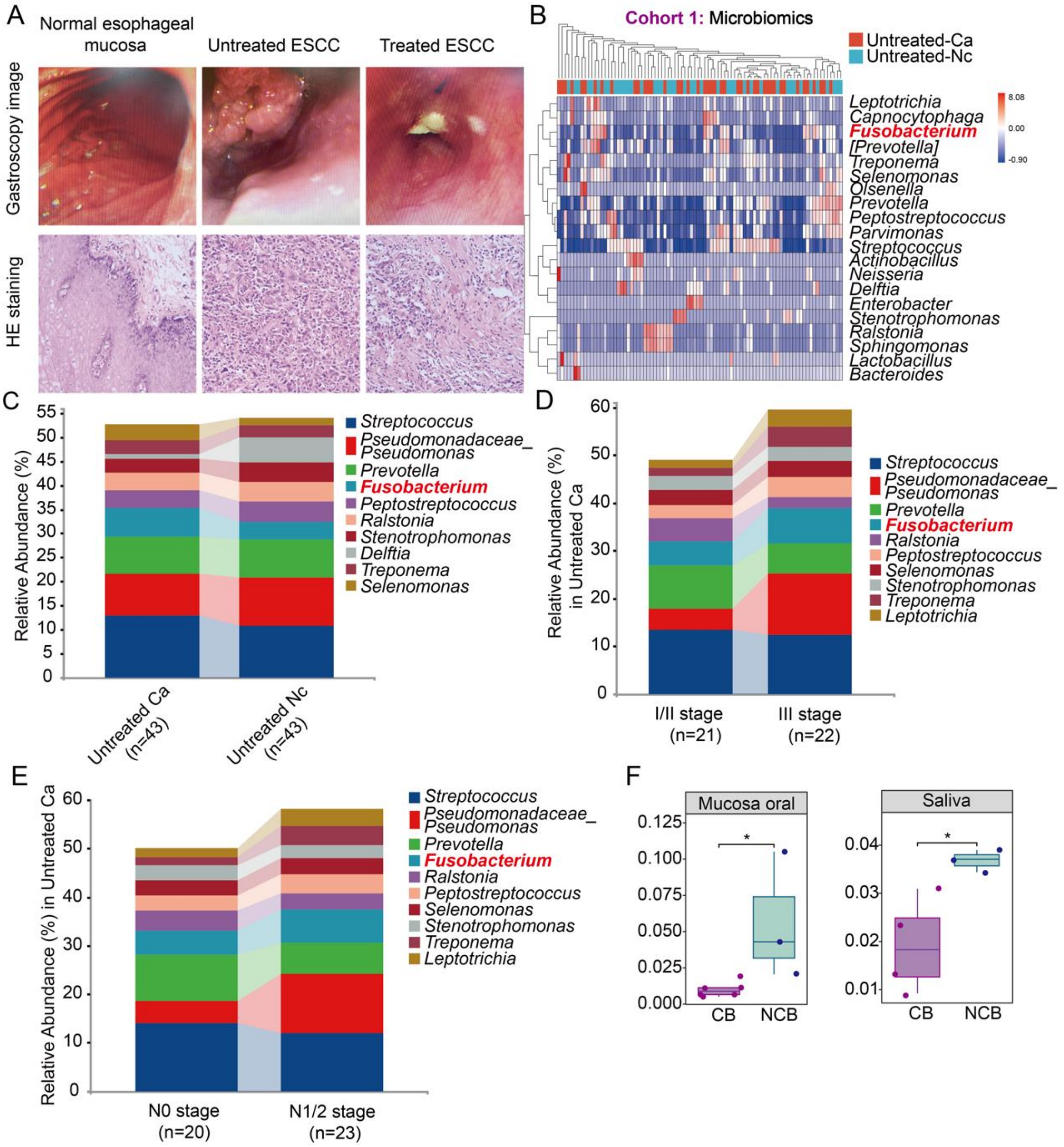

S-Fig. 1 Distribution of bacterial communities in ESCC tissues. (A) Preoperative gastroscopy images and corresponding postoperative hematoxylin-eosin (HE) staining images. (B-C) 16S rRNA microbiota analysis of cohort 1 at the genus level revealed distinct differences between untreated ESCC and paired normal tissues. (D-E) Species composition analysis of samples under different stages. (F) Correlation between the Fusobacterium genus in mucosa oral or saliva and the efficacy of immunotherapy in ESCC patients. The groups are clinical benefit (CB) group and no clinical benefit (NCB) Group. * $P<0.05$.

Supplementary figure 2

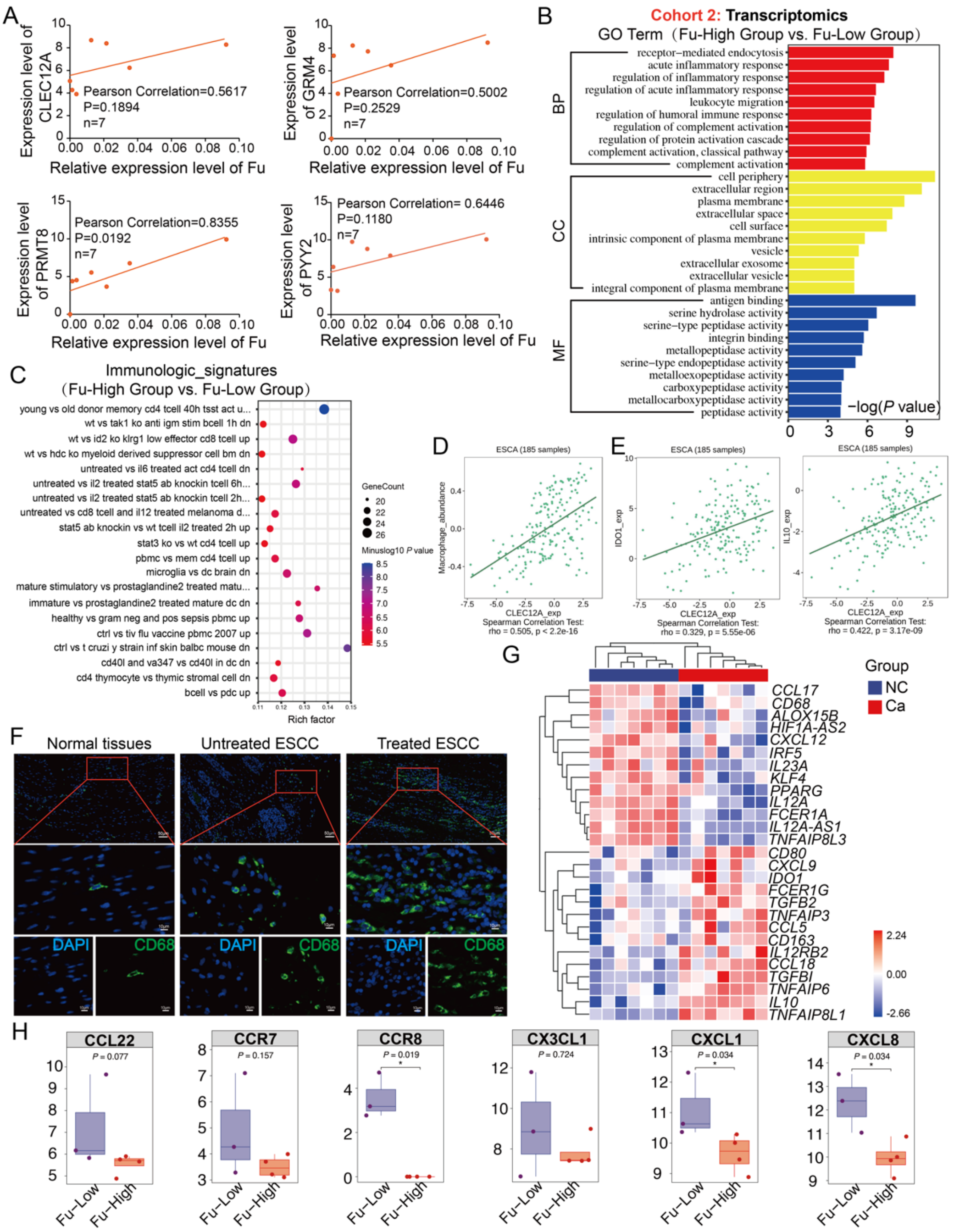

S-Fig. 2 Screening and functional identification of oncogenes associated with Fusobacterium. (A) A correlation analysis between Fusobacterium abundance and the target gene expression was conducted in ESCC samples. (B) Gene Ontology (GO) analysis on DEGs based on Fusobacterium abundance in ESCC tissues. (C) Human multi-database association annotation analysis about Immunologic signatures. (D-E) The relationship between CLEC12A and macrophages, as well as the respective genetic markers. (F) Immunofluorescence staining of CD68 in different tissues. (G) The heatmap of M1/M2 macrophage-associated genes in ESCC or paired normal tissues. (H) Joint analysis of chemokines and Fusobacterium abundance. * P<0.05.

Supplementary figure 3

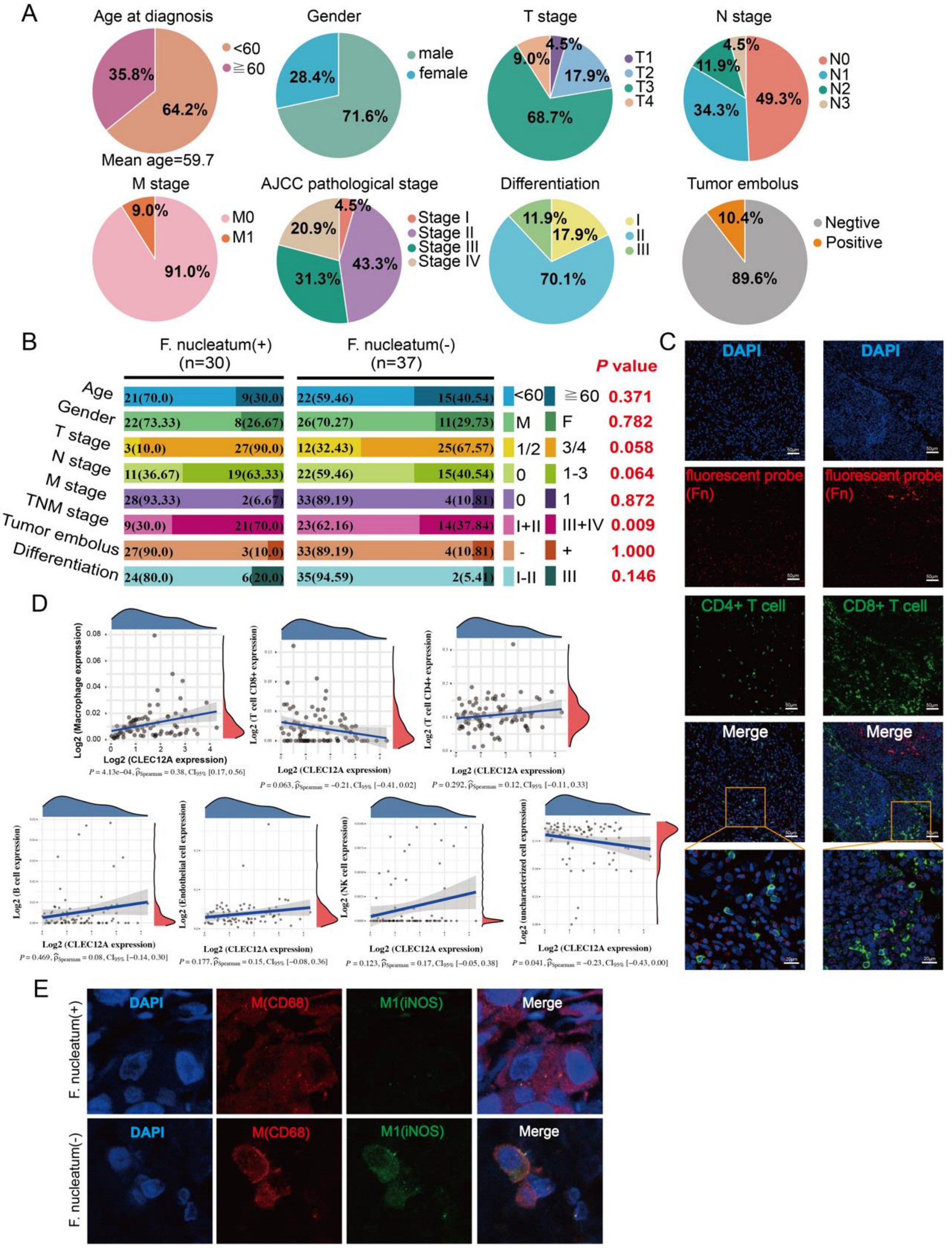

S-Fig. 3 Clinical data analysis for ESCC patients and the relationship between F. nucleatum and immune cells. (A) The clinical and pathological datas of the 67 ESCC patients in the gene chip. (B) The relationship between F. nucleatum infection and their clinical pathological characteristics. (C) Fluorescent probes of F. nucleatum and IF staining of immune cells on ESCC paraffin embedded specimens. (D) Database analysis between CLEC12A and different immune cells. (E) Dual IF of CD68 and iNOS in ESCC tissues with or without F. nucleatum infection.

Supplementary figure 4

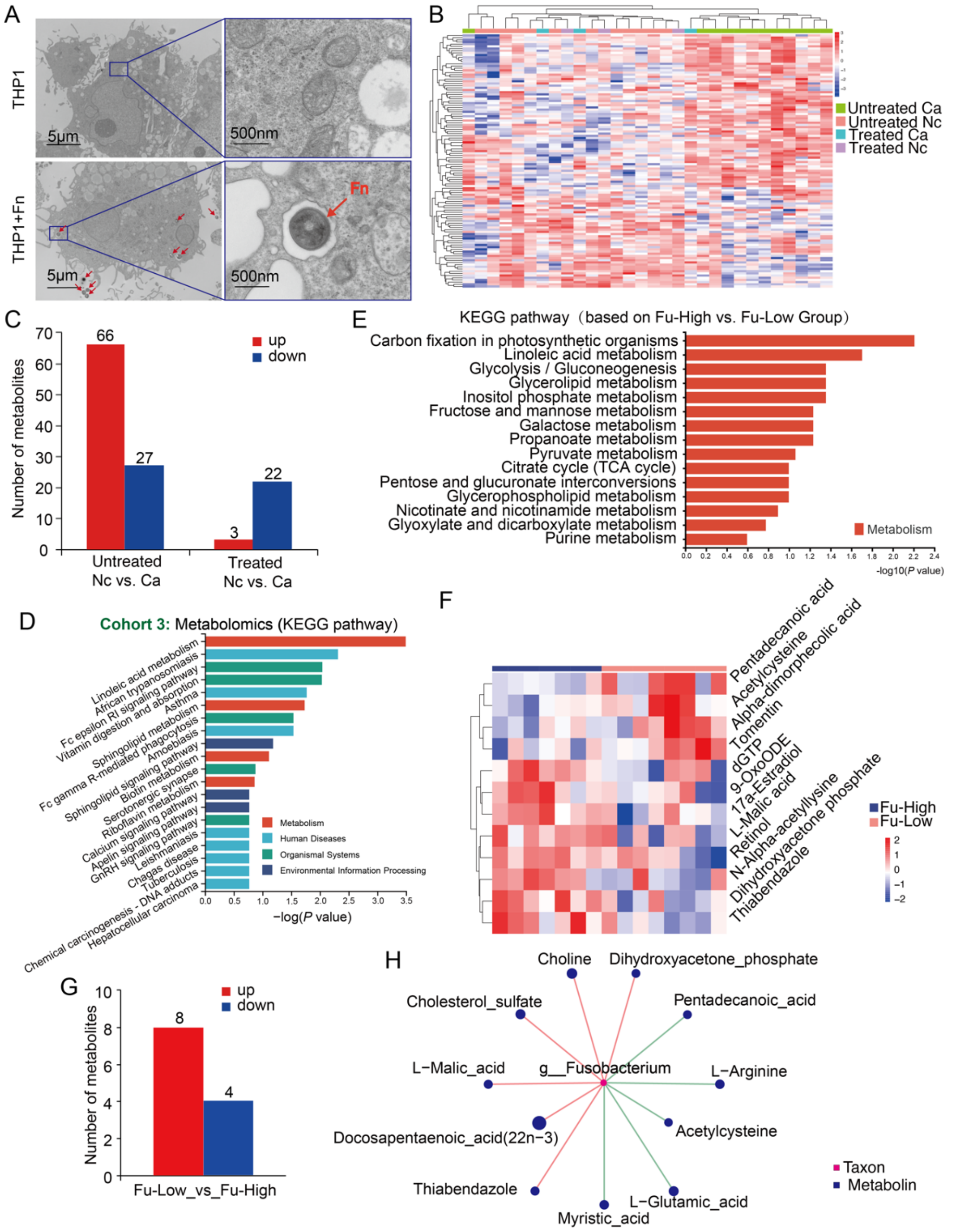

S-Fig. 4 Screening for metabolites related to Fusobacterium. (A) Electron microscopy analysis of samples from co-cultured THP1 macrophages with F. nucleatum. (B) Heatmap of Differential Metabolite Clustering Analysis between ESCC and normal tissues. (C, G) Selection of differential substances among different groups (Contrast: Control vs. Treatment). Statistical significance was assessed with a significance level set at *P*<0.05， fold-change(FC)>1 and VIP>1. (D-E) KEGG pathway enrichment analysis of differentially expressed metabolites. (F) Heatmap of Differential Metabolite Clustering Analysis based on F. nucleatum. (H) Combined analysis of metabolomics and microbiomics in 15 ESCC tissues revealed metabolites associated with Fusobacterium.

Supplementary figure 5

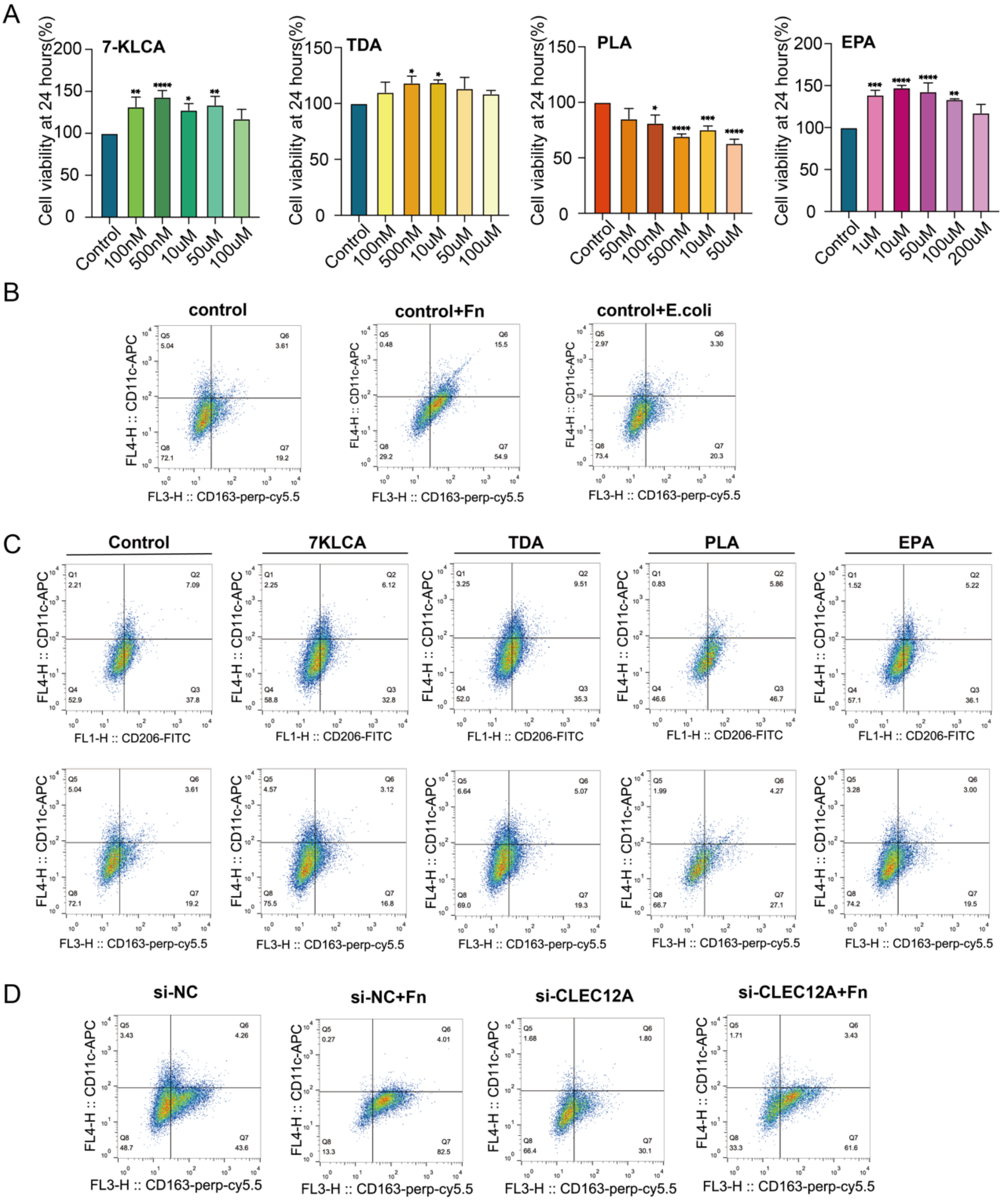

S-Fig. 5 The role of F. nucleatum, metabolites and CLEC12A on macrophage polarization. (A) The activity of THP1 macrophages changed following the interventions of metabolites. (B) Flow cytometry detection of changes in the distribution of macrophage CD11c and CD163 after co-culture with F. nucleatum or E. coli. (C) Flow cytometry detection of changes in the distribution of macrophage CD11c and CD206/CD163 after treated with different metabolites. (D) Flow cytometry detection of changes in the distribution of M1 and M2 macrophage in different groups. * $P<0.05$, ** $P<0.01$, *** $P<0.001$, **** $P<0.0001$.

Supplementary Table 2: The candidate metabolites associated with F. nucleatum by targeted metabolomics analysis

Name	HMDB	Cellular Location	Metabolite Levels in Different Groups (pg/ml)			
			Fn+KYSE-30	Nc+ KYSE-30	Fn+ECA109	Nc+ECA109
Tridecanoic acid (TDA)	HMDB0000910	Extracellular	2322.18	2125.485	4662.8325	2418.525
Myristelaidic acid (MA)	HMDB0062248	NA	168149.6634	—	190959.4101	—
Eicosapentaenoic acid (EPA)	HMDB0001999	Extracellular	45756.49456	—	56496.47919	—
2-Hydroxy-4-(methylthio)butanoic acid (HMTBa)	HMDB0037115	Cytoplasm	11.96704758	—	5.550706054	—
2-Hydroxycaproic acid (2-HCA)	HMDB0001624	NA	1550.048393	324.7426	1229.47223	153.4352
Glyceraldehyde (GA)	HMDB0001051	Cytoplasm	351138.1025	—	265624.5189	—
Kynurenine (KYN)	HMDB0000684	Cytoplasm	108156.0856	—	106879.0861	—
D-Arabinose (Ara)	HMDB0029942	Cytoplasm	584347779.9	—	430107295.3	—
7-Ketolithocholic acid (7-KLCA)	HMDB0000467	Extracellular	3208.265362	—	2054.309327	—
Benzenebutanoic acid (BBCA)	HMDB0000543	Membrane	146256.6807	—	103768.7748	—
Phenyllactic acid (PLA)	HMDB0000779	NA	3281.139955	419.7027	3006.776615	345.534
Phenol（PhOH）	HMDB0000228	NA	6473.655851	1559.996	21719.97416	4892.142

Supplementary table 3: The primer sequences of the genes used in this study

Gene	Forward	Reverse
CLEC12A	5’-ATTTTAGGGAGGCATGAGGC-3’	5’-CATGATGGAAAGTGGGAGGTG-3’
PRMT8	5’-TCCCCACCCCTTACCAGATGT-3’	5’-TGTGTGTTTATTGAGCGCCCA-3’
GRM4	5’-CCCTTTCCACAGTCATCCACC-3’	5’-TGCTACCCTCTCCCACCTCC-3’
F. nucleatum (NusG)	5’-AGTAGATCCTCGTGTATGGTATGAAG-3’	5’-TCCATAGGAATAGGGTCAGAACC-3’
GAPDH	5’-GTCTCCTCTGACTTCAACAGCG-3’	5’-ACCACCCTGTTGCTGTAGCCAA-3’
homo IL-6	5’-ACTCACCTCTTCAGAACGAATTG-3’	5’-CCATCTTTGGAAGGTTCAGGTTG-3’
homo TNF α	5’-GAGGCCAAGCCCTGGTATG-3’	5’-CGGGCCGATTGATCTCAGC-3’
homo NOS2	5’-TTCAGTATCACAACCTCAGCAAG-3’	5’-TGGACCTGCAAGTTAAAATCCC-3’
homo CXCL9	5’-CCAGTAGTGAGAAAGGGTCGC-3’	5’-AGGGCTTGGGGCAAATTGTT-3’
homo CXCL10	5’-GTGGCATTCAAGGAGTACCTC-3’	5’-TGATGGCCTTCGATTCTGGATT-3’
homo TGF-β1	5’-CTAATGGTGGAAACCCACAACG-3’	5’-TATCGCCAGGAATTGTTGCTG-3’
homo IL-4	5’-CCAACTGCTTCCCCCTCTG-3’	5’-TCTGTTACGGTCAACTCGGTG-3’
homo IL-10	5’-GACTTTAAGGGTTACCTGGGTTG-3’	5’-TCACATGCGCCTTGATGTCTG-3’
homo CD206	5’-GGGTTGCTATCACTCTCTATGC-3’	5’-TTTCTTGTCTGTTGCCGTAGTT-3’
homo CD163	5’-TTTGTCAACTTGAGTCCCTTCAC-3’	5’-TCCCGCTACACTTGTTTTCAC-3’
homo Arg1	5’-TGGACAGACTAGGAATTGGCA-3’	5’-CCAGTCCGTCAACATCAAAACT-3’

Materials and Methods

1 Tissue collection

ESCC tissues and paired normal tissues were obtained from 53 patients who underwent esophageal cancer resection at the Fourth Hospital of Hebei Medical University between July, 2018 and April, 2023. Immediately after the operation, the tissues were frozen in liquid nitrogen and stored at −80°C. The paraffin-embedded tissues and clinicopathological features were also obtained. At the time of specimen collection, all patients were free from any infections, and had not used antibiotics recently. This study was approved by the Ethical Review Boards of the Fourth Hospital of Hebei Medical University (Ethics Approval Number: 2023KS248). Informed consent was obtained from all the patients. In addition, we purchased cDNA chip (cDNA-HEsoS095Su01) containing 67 ESCC tissues and corresponding 28 adjacent normal tissues from Shanghai Outdo Biotech Co., Ltd (Shanghai, China).

2 Cell and Bacterial culture

KYSE-30, ECA-109 and THP1 cells were purchased from Type Culture Collection of the Chinese Academy of Science (Shanghai, China). The cells were authenticated using short tandem repeat (STR) profiling at the time of purchase. The F. nucleatum ATCC 25586 was purchased from the Guangdong Microbial Collection Center (Guangdong, China) and cultured at 37°C under anaerobic conditions.

3 16S rRNA Microbiomics

Total microbial genomic DNA samples were extracted using an OMEGA Soil DNA Kit (Omega Bio-Tek, GA, USA). The quantity and quality of extracted DNA were measured using a NanoDrop ND-1000 spectrophotometer (Thermo Fisher Scientific, MA, USA) and agarose gel electrophoresis, respectively. The V3–V4 regions of bacterial 16S rRNA genes were amplified by PCR using the forward primer

338F (5’-ACTCCTACGGGAGGCAGCA-3’) and the reverse primer 806R (5’-GGACTACHVGGGTWTCTAAT-3’). The sequencing platform was the Illumina MiSeq platform at Shanghai Personal Biotechnology Co., Ltd.

4 Nontargeted metabolomics

Accurately weigh an appropriate amount of tissue. Then add 1000 μL tissue extract [75%（9:1 methanol: chloroform):25% H_2O] and grind, repeat twice. Room temperature ultrasound for 30 min and Ice bath for 30 min. Centrifuge for 10 min at 12,000 rpm and 4°C, take all the supernatant, concentrate and dry. Add 200 μL 50% acetonitrile solution and prepared with 2-chloro-l-phenylalanine (4 ppm) to redissolve the sample, filter the supernatant by 0.22 μm membrane and transfer into the detection bottle for LC-MS detection. Identification and screen of differential metabolites were assisted by Shanghai Personal Biotechnology Co., Ltd. (Shanghai, China).

5 Targeted metabolomics of FFA

KYSE-30 and ECA-109 cells were divided into treated (co-cultured with F. nucleatum) and control groups. 0.05 mL of the supernatant was mixed with 150 μL MeOH, 200 μL MTBE, 50 μL 36% phosphoric acid/water (-20°C). Vortexed for 3 min (2500 r/min), centrifuged (12000 r/min, 5 min, 4°C). Took 200 μL supernatant, dried, added 300 μL 15% boron trifluoride methanol solution, vortexed (2500 r/min, 3 min), incubated (60°C, 30 min), cooled, added 500 μL n-hexane, 200 μL saturated sodium chloride solution. After vortexing (3 min) and centrifugation (4°C, 12000 r/min, 5 min), transferred 100 μL n-hexane layer for GC-MS analysis using the Agilent 7890B-7000D GC-MS/MS platform.

6 Targeted metabolomics of P650 project

400 μL of cold methanol/acetonitrile (1:1, v/v) extraction solvent was added to samples for protein removal and metabolite extraction. For absolute metabolite

quantification, stable-isotope internal standards were concurrently added to the extraction solvent. Centrifuged at 14000 g for 20 min at 4°C to collect the supernatant. The supernatant was then vacuum-dried. For LC-MS analysis, dried samples were re-dissolved in 100 μL acetonitrile/water (1:1, v/v), followed by centrifugation at 14000 g at 4 °C for 15 min, and the supernatant was injected. Analyses were conducted using a UHPLC (1290 Infinity LC, Agilent Technologies) coupled to a QTRAP MS (6500+, Sciex) at Shanghai Personal Biotechnology Co. Ltd.

7 Transcriptomics

Samples underwent RNA extraction with RNAmini kit (Qiagen, Germany). Strand-specific libraries were constructed using the TruSeq RNA sample preparation kit (Illumina, CA, USA), and sequencing was carried out using the Illumina Novaseq 6000 instrument. Raw data was processed using Skewer and assessed with FastQC v0.11.2. Reads (2×150 bp) were aligned to human genome hg38. The expression of the transcript was calculated by FPKM using Perl. Differentially expressed transcripts (DETs) were identified via MARS model (DEGseq package). The thresholds for determining DETs are $P<0.05$ and absolute fold change≥ 2. DETs were functionally analyzed using GO and KEGG databases, considering pathways with $P<0.05$ and at least two related genes. Screening range for DEGs was set as log2 Fold Change ≥ 3 when screening target genes of Fusobacterium.

8 qRT-PCR

Cells that have undergone treatment should be collected 24 hours post-treatment. Total RNA was extracted using TRIzol Reagent (Thermo, Waltham, MA), and reverse-transcribed into complementary DNA (cDNA) using the Reverse Transcription System (Promega, Fitchburg, WI). Then, the cDNA was subjected to quantitative PCR (qPCR) analysis using the 7500 RT-PCR System (AB Applied Biosystems). The comparative

Ct method (ΔCt) was used to analyze relative expression of genes. The primers used in the study were showed in Supplementary table 3.

9 Immunofluorescence (IF)

Deparaffinized tissue sections were placed in citrate buffer and boil in a microwave for 7 minutes. Then washed the sections with PBS 3 times, seal them with goat serum at 37°C for 1 hour, and then incubated the tissue sections with antibodies targeting CD68 (ab955, Abcam, Cambridge, UK), iNOS (MA5-17139, Invitrogen, California, USA), CD206 (60143-1-Ig, Protentech, Wuhan, China), CD4 (ab133616, Abcam, Cambridge, UK) or CD8 (ab217344, Abcam, Cambridge, UK), overnight at 4°C. After washing with PBS, incubated the tissue sections with the secondary antibody for 1 hour. Use DAPI to re dye the sample away from light for 20 minutes and fix it with an anti-fluorescence quenching reagent.

10 Fluorescent probe staining specific to F. nucleatum

Fix ESCC paraffin sections in 4% paraformaldehyde. Deparaffinize using methanol/xylene. Apply a F. nucleatum-specific DNA fluorescent probe, cover with a slip, and incubate for specific binding. Wash with PBS to remove unbound probes. Block nonspecific sites with bovine serum albumin, followed by nuclear staining. Observe under a fluorescence microscope, record F. nucleatum distribution and quantity. The specific probe sequence: 5'-CGCAATACAGAGTTGAGCCCTGC-3'.

11 Flow cytometry assay

THP-1 cells were induced with 100 ng/mL PMA (MultiSciences, China) for 48 h to polarize into M0 macrophages for subsequent experiments. Cells of different treatment groups were incubated with flow cytometry antibodies against CD11b and CD11c (TONBO Biosciences, California, USA), CD206 (BioLegend, California, USA),

and CD163(BioLegend, California, USA). The phenotypes of polarized cells were detected using flow cytometry.

12 ELISA

Collect the supernatant of THP1 cells of different groups (centrifuge at 1000×g, 10 min). Prepare various necessary solutions according to the manufacturer's instructions of Human IL-4 ELISA Kit, Human IL-6 ELISA Kit, Human IL-10 ELISA Kit and Human TNF-α ELISA Kit (Abclonal, Wuhan, China). Detect the OD values within 5 min after sample preprocessing. Fit the standard curve (R2 ≥ 0.99) and calculate the sample content.

13 Transient transfection

CLEC12A was silenced in THP1 macrophages with siRNAs (RIBOBIO, Guangzhou, China), according to the manufacturer's instructions. The target sequence is 5'GTCGTGAGCTATATAGCAA3'.

14 The predicted model of metabolite-protein binding

The structural models of PLA and CLEC12A protein were obtained from the PubChem and UniProt databases, respectively. The CLEC12A protein model was subjected to relaxation preprocessing using the Rosetta software, and the highest-scoring model was selected as the receptor for docking experiments. PLA was simulated in the physiological environment (pH 7.4) using the OpenBabel software. In PyMOL software, the library of conformers and receptor model were loaded, and initial binding coordinates between atoms were set. Docking experiments were performed for 100 conformers with the receptor using the Rosetta software, employing the RosettaLigand module within the RosettaScripts framework. A total of 20,000 docking attempts were conducted. Convergence screening of the generated structural library was performed for 4 rounds based on the total_score, and the highest-scoring model

was selected as the target model, considering the average values of each scoring term. Finally, the model was visualized using PyMOL software.

15 Transmission Electron Microscopy (TEM)

Cell samples were fixed in 2.5% glutaraldehyde for 2-4 hours at 4° C, washed thrice in 1/15M phosphate buffer, fixed in 1% osmium tetroxide for 1-2 hours, and washed three more times. They were then dehydrated in graded acetone (50%, 70%, 80%, 90%) for 10-15 minutes each, followed by two 10-15 minutes washes in 100% acetone. The cell samples were infiltrated, embedded, polymerized, and sectioned into 50 nm slices using an ultramicrotome (Leica UC-7). They were then stained with uranyl acetate and lead citrate and observed with a transmission electron microscope (Hitachi H-7500, Japan).

16 Statistical analyses

Differences between groups were evaluated with two-tailed Student's t-tests. The correlation of categorical variables was analyzed using the chi-square test. Spearman's correlation analysis was used to describe the correlation between quantitative variables without a normal distribution. Linear discriminant analysis effect size (LEfSe) analysis was performed to assess taxonomic differences between the two groups. Almost each cell experiment was repeated at least three times. Statistical analyses were performed using GraphPad Prism software (Inc:La Jolla, CA, USA), SPSS software 21.0 (SPSS Inc., USA) and R v4.0.3. $P<0.05$ was considered as statistically significant. *$P<0.05$, ** $P<0.01$, *** $P<0.001$, **** $P<0.0001$.
